# Occupational Stress among Field Epidemiologists in Field Epidemiology Training Programs from the Public Health Sector

**DOI:** 10.3390/ijerph16183427

**Published:** 2019-09-16

**Authors:** Sukhyun Ryu, Young Woo Kim, Seowon Kim, Qiuyan Liao, Benjamin J. Cowling, Chang-Seop Lee

**Affiliations:** 1 WHO Collaborating Centre for Infectious Disease Epidemiology and Control, School of Public Health, Li Ka Shing Faculty of Medicine, The University of Hong Kong, Hong Kong, China; gentryu@hanmail.net (S.R.); bcowling@hku.hk (B.J.C.); 2 Department of Preventive Medicine, College of Medicine, Konyang University, Daejeon 35365, Korea; 3 Department of Epidemiology and Health Informatics, Graduate School of Public Health, Korea University, Seoul 02841, Korea; entristecer@naver.com (Y.W.K.); kimseo6@hanmail.net (S.K.); 4 Division of Behavioural Sciences, Li Ka Shing Faculty of Medicine, The University of Hong Kong, Hong Kong, China; qyliao11@hku.hk; 5 Department of Internal Medicine, Chonbuk National University Medical School, Jeonju 54907, Korea; 6 Research Institute of Clinical Medicine of Chonbuk National University-Biomedical Research Institute of Chonbuk National University Hospital, Jeonju 54907, Korea

**Keywords:** occupational stress, field epidemiologist, burnout, trainee, training

## Abstract

Despite the high-demand work environment for field epidemiologists in field epidemiology training programs, little is known about their occupational stress. To identify occupational stress and its related factors, the occupational stress among trainees in field epidemiology training programs in Southeast Asia and Western Pacific regions from 2016 to 2018 was examined using six subscales: Role Overload, Role Insufficiency, Role Ambiguity, Role Boundary, Responsibility, and Physical Environment. Furthermore, the data on the year of training and type of training program as well as the level of burnout, which affects stress-coping strategies, were collected. Fisher’s exact tests and logistic regression models were used to examine associations between occupational stress, burnout, the number of years of training, and the type of training program. Sixty-two trainees participated, and there were no significant associations between burnout, the year of training, and type of training program. A burden of occupational stress in Role Overload and Physical Environment was reported by 56% and 53% of respondents, respectively. The trainees affiliated with a university program were less likely to have a burden of occupational stress in Responsibility and Physical Environment. It is concerning that more than half of trainees in the programs experienced occupational stress in Role Overload and Physical Environment. Additional efforts to design improved training programs to reduce occupational stress are warranted.

## 1. Introduction

Occupational stress is defined as harmful physical and emotional responses acquired from the working environment [1]. It is associated with burnout, which has been defined as a syndrome of emotional exhaustion, depersonalization, and a weakened sense of personal accomplishment, which is related to personal characteristics including passive-aggressive traits and depression [2,3,4]. Occupational stress presents in all occupations [5], and excessive occupational stress leads to reduce work performance and increase staff turnover [6].

Field epidemiologists, the frontline public health workforce, respond to outbreaks of communicable diseases, conduct routine public health activities, as well as respond to other outbreaks and emergencies [7,8]. Many countries have initiated Field Epidemiology Training Programs (FETPs) to train field epidemiologists in public health surveillance and outbreaks response [9]. FETPs are usually coordinated and taught by public health agencies, and in some countries by universities [10,11]. However, many countries have difficulties in attracting and retaining field epidemiologists, because of high turnover rates caused by high-responsibility and the principle of learning-by-doing which is the key element of FETPs [8,12,13,14]. Furthermore, limited academic resources, including the clinically-oriented training program which is population-based, as well as a lack of proper teaching methodologies lead trainees to have insufficient opportunities for professional development to respond to public health emergencies [15].

Despite the high-demand work environment for field epidemiologists, little is known about their occupational stress, burnout, or about the impact of the format of training program, especially among less experienced trainees. The purpose of this study was to explore the level of occupational stress and burnout among FETP trainees, and identify differences based on the type of their training program (university-affiliated or not). The present study may be useful to improve FETPs, enrich the workforce in the public health sector, and enhance national health security.

## 2. Materials and Methods

This was a cross-sectional study using written questionnaires given to FETP trainees from countries in South Asia and Western Pacific regions in the cohort of years 2016 to 2018. An anonymous self-administered questionnaire was used to measure the level of occupational stress and burnout. The participants were volunteers recruited as a convenience sample during the Southeast Asia and Western Pacific bi-regional Training Programs in Epidemiology and Public Health Interventions Network (TEPHINET) Conference at Vientiane, Laos, between 5 and 9 November, 2018. Two hundred and twenty-two public health experts, including field epidemiologists, participated [16].

The information from each respondent on the year of their training, FETP country, and training program affiliations (e.g., university or government) was collected. The level of burnout was measured to use as a proxy of individual traits including educational level, working hours, and personality characteristics that can affect occupational stress-coping strategies [3,17,18,19]. The Maslach Burnout Inventory—Human Services Survey (MBI—HSS), which is the leading instrument for the assessment of burnout, was used. MBI—HSS measures the three dimensions of burnout: Emotional Exhaustion, which measures sentiments of being emotionally exhausted by the respondent’s work (8 items); Depersonalization, which assesses impersonal responses toward the recipients of the respondent’s work (5 items); and Personal Accomplishment, which measures feelings of achievement in the respondent’s work (7 items). A higher score for Emotional Exhaustion and Depersonalization and a lower score for Personal Accomplishment is associated with a higher tendency of burnout in respondents [20]. Total scores on each dimension of burnout were stratified into high, moderate, or low tertiles using cutoffs for each tertile based on previously validated normative MBI—HSS data for social services (Emotional Exhaustion: low <11, moderate 11 to 31, high ≥32; Depersonalization: low <2, moderate 2 to 12, high ≥13; and Personal Accomplishment: low ≥41, moderate 26 to 40, high ≤25) [17,20]. Overall burnout was classified by satisfying criteria for burnout in any two dimensions or in all dimensions [20]. In the present study, the values for Cronbach’s α were 0.85 for Emotional Exhaustion, 0.86 for Depersonalization, and 0.87 for Personal Accomplishment, indicating that the internal consistency of the scales was generally reliable [21].

Occupational stress was measured using the Occupational Roles Questionnaires (ORQ) derived from the Occupational Stress Inventory—Revised edition (OSI-R), which was concise and well-validated in a previous study [22]. The ORQ comprises measures of six subscales, including Role Overload, Role Insufficiency, Role Ambiguity, Role Boundary, Responsibility, and Physical Environment, each including 10 items. A response scale from each item ranged from 1 (never) to 5 (often) and a higher score of each subscale indicates more occupational stress. Total raw scores of the six subscales were transformed to a T score, which was based on a normative sample, and then based on the T score, it was stratified into three levels: maladaptive occupational stress (T ≥ 60), normal range (T 40 to 59), or absence of occupational stress (T < 40) [23]. A detailed interpretation of the high score of the six subscales is described in Table 1. In the present study, the values of Cronbach’s α were 0.86 for Role Overload, 0.87 for Role Insufficiency, 0.86 for Role Ambiguity, 0.86 for Role Boundary, 0.86 for Responsibility, and 0.86 for Physical Environment. This indicated that the subscales had good internal consistency [21].

Fisher’s exact tests were used to identify associations between demographic variables, including the year of training, the type of the training program, the level of occupational stress, and burnout. Logistic regression models were used to further identify factors independently associated with occupational stress. All statistical analyses were performed using R version 3.2.4 (R Foundation for Statistical Computing, Vienna, Austria). All statistical tests were two-sided and a *p*-value < 0.05 was considered statistically significant.

This study was approved by the ethics committee of the Institutional Review Board designated by Chonbuk National University (JBNU-10-006). Written informed consent was obtained from all study participants.

## 3. Results

Overall, 64 FETP trainees participated, and a total of 62 trainees completed the survey (response rate: 97%) between 5 and 9 November, 2018. Participants were drawn from 14 different countries, including Australia (number of participants: 3, proportion of total survey participants: 4.8%), Bangladesh (1, 1.6%), China (9, 14.5%), India (11, 17.5%), Indonesia (7, 11.3%) Laos (2, 3.2%), Malaysia (4, 6.5%), Myanmar (2, 3.2%), Philippine (7, 11.3%), Singapore (2, 3.2%), Taiwan (1, 1.6%), Thailand (5, 8.1%), Vietnam (7, 11.3%), and Japan (1, 1.6%). The length of working years in FETPs was classified as less than a year (3, 4.8%), 1 to less than 2 years (13, 21%), 2 to less than 3 years (38, 61.3%), and 3 years or above (8, 12.9%).

**Figure 1:**
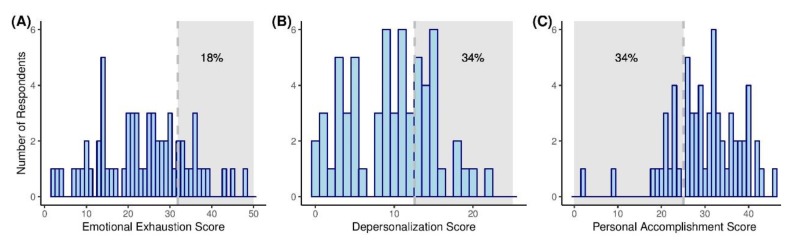
Maslach Burnout Inventory—Human Services Survey subscale scores. Histograms depicting score frequency by subdomains of (**A**) Emotional Exhaustion, (**B**) Depersonalization, and (**C**) Personal Accomplishment. The number of percentage in the shaded region indicates the proportion of the highest tertile in each domain. Overall burnout was considered present when any two or all domains met the criteria (Emotional Exhaustion ≥ 32, Depersonalization ≥ 13, or Personal Accomplishment ≤ 25).

The result of univariate analysis between the demographic variables, burnout, and occupational stress is shown in Table 2. Among the respondents, 18% (*n* = 11), 34% (*n* = 21), and 23% (*n* = 14) scored in the highest Emotional Exhaustion and Depersonalization tertile, and in the lowest Personal Accomplishment tertile, respectively (Figure 1). A total of 19% (*n* = 12) satisfied the requirement for overall burnout. However, no significant differences were identified between burnout and the year of training, or the type of training program.

**Figure 2:**
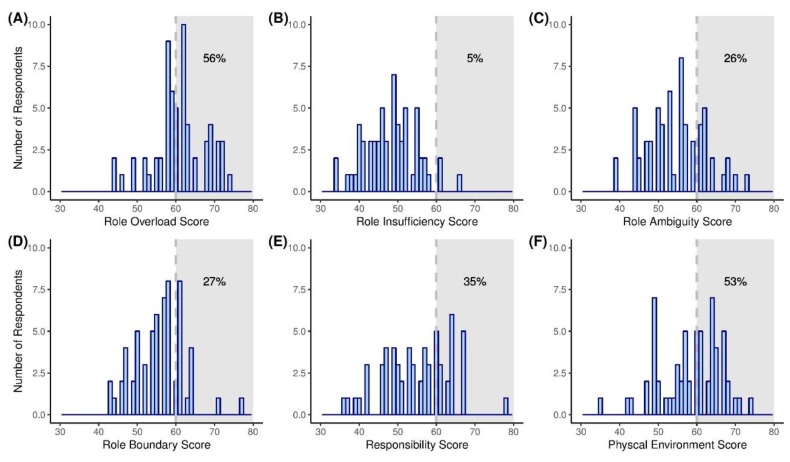
Occupational role scores from the Occupational Stress Inventory—Revised edition. Histograms depicting score frequency by the subdomains of (**A**) Role Overload, (**B**) Role Insufficiency, (**C**) Role Ambiguity, (**D**) Role Boundary, (**E**) Responsibility, and (**F**) Physical Environment. Shaded regions which T scores greater than 60 indicate the presence of a burden of occupational stress in each subdomain.

Based on OSI-R normative data, 57% (*n* = 35), 5% (*n* = 3), 26% (*n* = 16), 27% (*n* = 17), 36% (*n* = 22), and 53% (*n* = 33) of respondents had maladaptive levels of occupational stress in the subdomains of Role Overload, Role Insufficiency, Role Ambiguity, Role Boundary, Responsibility, and Physical Environment, respectively (Figure 2). Among respondents, 54% of first-year trainees had a burden of occupational stress in Role Overload, and more than half of the second-year trainees had a burden of occupational stress in Role Overload (*n* = 25, 66% of second-year trainees), Role Ambiguity (*n* = 9, 56%), Role Boundary (*n* = 10, 59%), Responsibility (*n* = 17, 77%), and Physical Environment (*n* = 23, 70%). However, there was no significant difference between the year of training and the level of occupational stress in all subdomains. According to the type of training program, 60% (*n* = 31) and 63% (*n* = 33) of trainees affiliated with government programs had occupational stress in Role Overload and Physical Environment. In contrast, trainees affiliated with a university did not have a burden of stress in Role Insufficiency, Responsibility, and Physical Environment. The occupational stress in Responsibility and Physical Environment of university-affiliated trainees was significantly lower (*p* = 0.01, *p* < 0.01, respectively) than other trainees.

The result of logistic regression analysis to examine independent associations between the binary outcomes of occupational stress in each subdomain and variables of interest is shown in Table 3. First- and second-year trainees were more likely to have a burden of stress in Role Overload (odds ratio = 1.91, 95% Confidence Interval = 1.03–1.66 for first-year trainees; and 2.05, 95% CI = 1.16–3.62 for second-year trainees). The trainees with overall burnout were more likely to have stress in Role Boundary (odds ratio = 1.51, 95% CI = 1.16–1.97). However, trainees affiliated with a university were less likely to have a burden of occupational stress in the domains of Responsibility and Physical Environment (odds ratio = 0.65, 95% CI = 0.48–0.90; and 0.54, 95% CI = 0.39–0.73).

## 4. Discussion

Reducing occupational stress is crucial for improving work performance and reducing job turnover [24]. It is alarming that, in the present study, more than half of the trainees in FETPs experienced occupational stress from Role Overload and Physical Environment.

Across the several studies [25,26,27], the prevalence of burnout in general population ranges from 13% to 27%, indicating that a moderate portion of trainees in our study population experienced burnout. There was no significant difference between burnout and year of training, which is consistent with a previous study on burnout in the training of medical professionals [28]. Furthermore, there was no significant difference between burnout and the type of program. An earlier study demonstrated that training and practicing in academic settings were less likely to experience burnout [29]; however, trainee-affiliated government agencies might be less likely to experience burnout as well, because they generally have greater flexibility in scheduling and a more stable job status than trainees in academic settings [30].

Occupational stress in each domain occurred in approximately 15–20% of the normative sample among public service and safety employees, indicating that the respondents in our study population felt more burdened with considerable role overload and undesirable physical work conditions than the normative sample population [23]. High scores of occupational stress in the subdomains of Role Overload and Physical Environment were not surprising, given that field epidemiologists must exhibit a rapid response to disease outbreaks, requiring decision-making with potentially serious consequences and potential exposure to hazardous field sites [8,31]. The burden of stress from Role Overload was particularly higher in the trainees of first and second years, likely because the trainees have more work compared to new trainees and have less experience in responding to the work compared to trainees of more than 2 years. This finding is corroborated by a previous study showing that senior trainees in medical professions had lower occupational stress, which was positively correlated with workload [32]. In the subdomain of Responsibility and Physical Environment, we identified differences in trainees’ occupational stress burden based on whether their training program was university-affiliated or government-affiliated. Furthermore, the results from the logistic regression analysis demonstrated that the type of training program was associated with occupational stress in the subdomain of Responsibility and Physical Environment, where university-affiliated trainees had a lower burden of occupational stress in both subdomains. This is likely because academic-affiliated programs provide a structure that supports trainees with a professional advisory group which may help trainees with appropriate guidance for immediate response in challenging situations [11]. Furthermore, training in an academic institution where there is a favorable environment of academic support may reduce the burden of stress in the domain of the working environment [11].

The burden of stress on Role Boundary was higher among trainees experiencing overall burnout. However, given that unclear role boundaries may affect the level of individual burnout [33], additional studies are needed to understand this finding.

Reducing occupational stress improves the work quality and productivity of employees [34]; therefore, it can facilitate sustainable development of a future public health workforce. To improve trainees’ capabilities, TEPHINET has developed standardized training accreditation criteria, including mentorship programs and academic support [35]; however, few member countries are accredited. Some countries have made efforts to improve their FETPs through the evaluation of the programs in terms of sustainability and academic quality assurance [11,31]. However, several challenges are still remaining. First, the trainees of FETPs commonly encounter the difficulty of finding the qualified and experienced epidemiologist to serve as a mentor [8,11]. Second, unsteady technical and academic support to FETPs may limit the sustainability of the program [8,11]. Therefore, tying FETPs with academic settings is needed not only to reduce the occupational stress of the individual trainee but also to enhance the strong mentorship to FETPs and their sustainability.

The findings of the present study should be interpreted in light of some limitations. First, the respondents of this survey did not provide a representative sample of field epidemiology trainees in all regions. In addition, the occupational stress of individuals can be affected by personal characteristics and demographic factors, such as younger age, personality traits, level of education, income, and weekly working hours, which we did not measure [5,36]. However, to account for differences in demographic factors that might affect the level of occupational stress, the level of burnout was measured to use as a proxy of individual traits [37]. Second, participants who attended the conference were likely to be self-motivated individuals, and there may be differences between respondents and non-respondents. Third, data obtained through self-reported questionnaires have a potential bias due to the reliance on self-reporting. Although these biases likely affected our results, our study can guide implementation of larger-scale studies of FETPs. Furthermore, our findings are of importance for FETP training resource development, by exploring trainee occupational stress and providing information for the management of the occupational environment.

## 5. Conclusions

In this study, trainees of field epidemiologists in university-affiliated training programs had lower occupational stress in terms of responsibility and the physical environment. Additional studies to identify at-risk populations and design improved training programs, such as embedding with a university to reduce occupational stress, are warranted.

## Figures and Tables

**Table 1:** High score interpretation of the Occupational Role Questionnaire [23].

Subdomain	Interpretation
Role Overload	A high score suggests their workload as increasing and not receiving appropriate support. The respondents with high score may not be well trained or not have enough knowledge for the job.
Role Insufficiency	A high score indicates their skills are unsuitable for their job. The respondents with high score may present a lack of interest in their work.
Role Ambiguity	A high score suggests an ambiguous feeling of what they are expected to do on their job. The respondents with high score may not present the clear aim of their work.
Role Boundary	A high score indicates conflicting sense captured between demands from supervisor and factions. The respondents with high score may have ambiguous about the authority line.
Responsibility	A high score suggests a high level of responsibility for their work. The respondents with high score tend to seek out for leadership and to have pressure from colleagues or the public.
Physical Environment	A high score indicates respondents are likely to be exposed to the high level of noise or unpleasant situation. The respondents with high score may have unpredictable work schedule or feel solitary.

**Table 2:** Study sample and variables associated with occupational stress and burnout in univariate analysis.

Burnout and Occupational Stress		Year of Trainee					Program Type		
		<1	1	2	3	*p*-Value	Government	University	*p*-Value
	Total	3 (5%)	13 (21%)	38 (61%)	8 (13%)		52 (84%)	10 (16%)	
**Burnout subscale**									
Emotional Exhaustion	11	0	2 (11%)	8 (21%)	1 (13%)	1.00	9 (17%)	2 (20%)	1.00
Depersonalization	21	3 (100%)	4 (31%)	12 (32%)	2 (25%)	1.00	18 (35%)	3 (30%)	1.00
Personal Accomplishment	14	1 (33%)	1 (8%)	10 (26%)	2 (25%)	0.68	11 (21%)	3 (30%)	0.68
Overall Burnout	12	1 (33%)	2 (11%)	8 (21%)	1 (13%)	1.00	10 (19%)	2 (20%)	1.00
**Occupational stress subscale**									
Role Overload	35	0	7 (54%)	25 (66%)	3 (38%)	0.09	31 (60%)	4 (40%)	0.31
Role Insufficiency	3	0	1 (8%)	1 (3%)	1 (13%)	0.39	3 (6%)	0	1.00
Role Ambiguity	16	2 (67%)	3 (23%)	9 (24%)	2 (25%)	0.46	12 (23%)	4 (40%)	0.27
Role Boundary	17	0	4 (31%)	10 (26%)	3 (38%)	0.75	16 (31%)	1 (10%)	0.26
Responsibility	22	1 (33%)	2 (15%)	17 (45%)	2 (25%)	0.23	22 (42%)	0	0.01 *
Physical Environment	33	2 (67%)	4 (31%)	23 (61%)	4 (50%)	0.30	33 (63%)	0	<0.01 *

* significant.

**Table 3:** Ordinary logistic regression of variables associated with occupational stress in each subdomain.

Variables	Role Overload	Role Insufficiency	Role Ambiguity	Role Boundary	Responsibility	Physical Environment
	Odds Ratio (95% CI)	Odds Ratio (95% CI)	Odds Ratio (95% CI)	Odds Ratio (95% CI)	Odds Ratio (95% CI)	Odds Ratio (95% CI)
**Year of training**						
<1	1.00	1.00	1.00	1.00	1.00	1.00
1	1.91 (1.03–1.66) *	1.11 (0.84–1.47)	0.64 (0.37–1.10)	1.58 (0.92–2.70)	0.98 (0.55–1.76)	0.87 (0.49–1.54)
2	2.05 (1.16–3.62) *	1.04 (0.80–1.35)	0.65 (0.40–1.08)	1.42 (0.86–2.34)	1.22 (0.71–2.10)	1.06 (0.62–1.80)
3	0.50 (0.79–2.84)	1.15 (0.85–1.54)	0.70 (0.40–1.24)	1.59 (0.90–2.78)	0.95 (0.52–1.75)	0.87 (0.48–1.59)
**Program type**						
Government affiliated	1.00	1.00	1.00	1.00	1.00	1.00
University affiliated	0.76 (0.54–1.07)	0.94 (0.81–1.10)	1.23 (0.91–1.66)	0.79 (0.59–2.78)	0.65 (0.48–0.90) *	0.54 (0.39–0.73) *
**Overall burnout**						
No	1.00	1.00	1.00	1.00	1.00	1.00
Yes	1.15 (0.85–1.56)	1.06 (0.92–1.21)	1.33 (1.01–1.74) *	1.51 (1.16–1.97) *	1.18 (0.88–1.58)	1.16 (0.88–1.55)

CI: Confidence Interval. * significant.
